# Voice-based control system for smart hospital wards: a pilot study of patient acceptance

**DOI:** 10.1186/s12913-022-07668-1

**Published:** 2022-03-03

**Authors:** Wen-Shan Jian, Ju-Yu Wang, Annisa Ristya Rahmanti, Shuo-Chen Chien, Chun-Kung Hsu, Chia-Hui Chien, Yu-Chuan Li, Chun-You Chen, Yen-Po Chin, Chen-Ling Huang

**Affiliations:** 1 School of Health Care Administration, Taipei Medical University, Taipei, Taiwan; 2 School of Gerontology Health Management, College of Nursing, Taipei Medical University, Taipei, Taiwan; 3 Research Center for Artificial Intelligence in Medicine, Taipei Medical University, Taipei, Taiwan; 4 Department of Nursing, Wan Fang Hospital, Taipei Medical University, Taipei, Taiwan; 5 Graduate Institute of Biomedical Informatics, College of Medical Science and Technology, Taipei Medical University, Taipei, Taiwan; 6 International Center for Health Information and Technology, College of Medical Science and Technology, Taipei Medical University, Taipei, Taiwan; 7 Information Technology Office, Wan Fang Hospital, Taipei Medical University, Taipei, Taiwan; 8 Department of Dermatology, Wan Fang Hospital, Taipei Medical University, Taipei, Taiwan; 9 Department of Radiation Oncology, Wan Fang Hospital, Taipei Medical University, Taipei, Taiwan; 10 Department of Biomedical Informatics, Harvard Medical School, Boston, USA; 11 Department of Medicine, Brigham and Women’s Hospital and Harvard Medical School, Boston, USA; 12 Division of Endocrinology and Metabolism, Department of Internal Medicine, Taipei Medical University Hospital, Taipei, Taiwan

**Keywords:** Technology acceptance model (TAM), Voice-based control system, Patient autonomy, Smart hospital

## Abstract

**Background:**

The smart hospital's concept of using the Internet of Things (IoT) to reduce human resources demand has become more popular in the aging society.

**Objective:**

To implement the voice smart care (VSC) system in hospital wards and explore patient acceptance via the Technology Acceptance Model (TAM).

**Methods:**

A structured questionnaire based on TAM was developed and validated as a research tool. Only the patients hospitalized in the VSC wards and who used it for more than two days were invited to fill the questionnaire. Statistical variables were analyzed using SPSS version 24.0. A total of 30 valid questionnaires were finally obtained after excluding two incomplete questionnaires. Cronbach’s α values for all study constructs were above 0.84.

**Result:**

We observed that perceived ease of use on perceived usefulness, perceived usefulness on user satisfaction and attitude toward using, and attitude toward using on behavioral intention to use had statistical significance (*p* < .01), respectively.

**Conclusion:**

We have successfully developed the VSC system in a Taiwanese academic medical center. Our study indicated that perceived usefulness was a crucial factor, which means the system function should precisely meet the patients' demands. Additionally, a clever system design is important since perceived ease of use positively affects perceived usefulness. The insight generated from this study could be beneficial to hospitals when implementing similar systems to their wards.

**Supplementary Information:**

The online version contains supplementary material available at 10.1186/s12913-022-07668-1.

## Introduction

The increased demand for healthcare services, particularly in an aging society, has become a major challenge in developed countries. Previous studies have proposed that governments and healthcare providers such as hospitals and long-term care institutions should prepare a coping solution to ensure comprehensive care [1, 2]. Intuitively, increasing the amount of medical staff to guarantee that every demand could be satisfied is the most straightforward way. However, human resources are precious. Therefore, using information technology to reduce human resources and improve nursing efficiency has become a crucial issue.

Voice-based control, a specific IoT technology application used in healthcare environments, could improve healthcare quality and experience [3–5]. The voiced-based control system was also an effective way to reduce contamination of surfaces, which could decrease the spread of healthcare-associated infections (HCAIs) with touchless computer interfaces [5]. Not only beneficial for preventing nosocomial infection, but the voice-based control system is also suitable for all hospitalized patients and helpful for post-surgery individuals [6]. Patients can lie on the ward bed and control the facilities in the room without contact, making the hospitalization more affable for mobility patients.

Since patients are consumers under this condition, studying their points before the voice-based control system's actual implementation is essential. Previous studies conducted in laboratory environments showed that users considered positive with different interaction modes in the wards [3, 6]. Here, we conducted an experimental study in the real clinical workflow. The technology acceptance model (TAM), a robust theory that systematically explains why users accept or reject new technology, has been widely used in many previous studies [7]. The TAM constructs (e.g., perceived ease of use, perceived usefulness, and behavioral intention to use) could further determine the key factor of whether people adopt a voice-based control system. The result could provide valuable insights into increasing patient satisfaction when healthcare providers design this kind of system.

Thus, in this study, a structural questionnaire adopting TAM was used to evaluate the patient acceptance of the voice-based control system implemented in the real clinical workflow of academic medical center wards. Finally, we discuss the advantages of using the VSC system and identify potential opportunities for the hospital when building similar systems.

## Materials and Methods

The study was conducted in a Taiwanese academic medical center between January 15, 2019, and September 4, 2019. A structural questionnaire was used as a research tool. We only included patients with behavioral capacity and stayed in the VSC ward for at least two days. For the mobility patients, their caretakers, such as parents or family members, helped those who were not able to complete the questionnaire to fill in. The questionnaire was given to patients and was filled onsite one day before the discharge. The incompletely filled questionnaire was excluded.

### System design and implementation– voice smart care (VSC) system

**Fig. 1:**
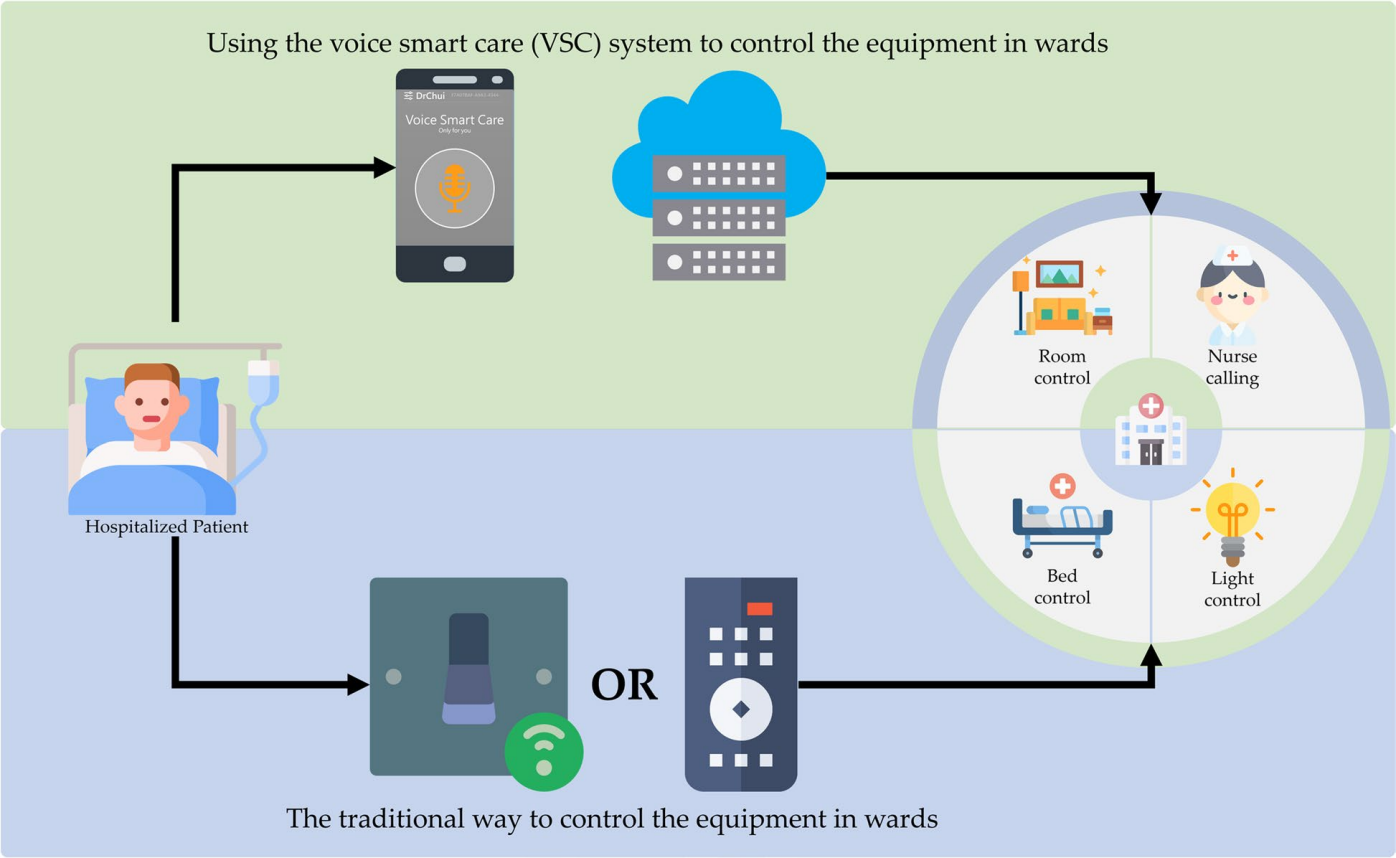
Different ways to control ward facilities

Traditionally, patients control the equipment in the ward mainly through switches, controllers, or assistance from other people (Fig. 1). They need to use the corresponding way to control the wards' facilities. The solution of integration control was lacking. Thus, the VSC system, a novel approach that allows patients to control ward facilities through their own mobile devices, was implemented in this study.

In order to achieve the purpose mentioned above, retrofit to the original ward facilities was implemented to what would be controlled by the VSC system. External hardware "smart switch module", was added to make these facilities were available controlled by the Wi-Fi signals. The original control way has been retained at the same time. Two VSC wards were retrofitted from general pediatric wards, took two weeks to complete construction, not limited to the children and adults for the move-in.

**Fig. 2:**
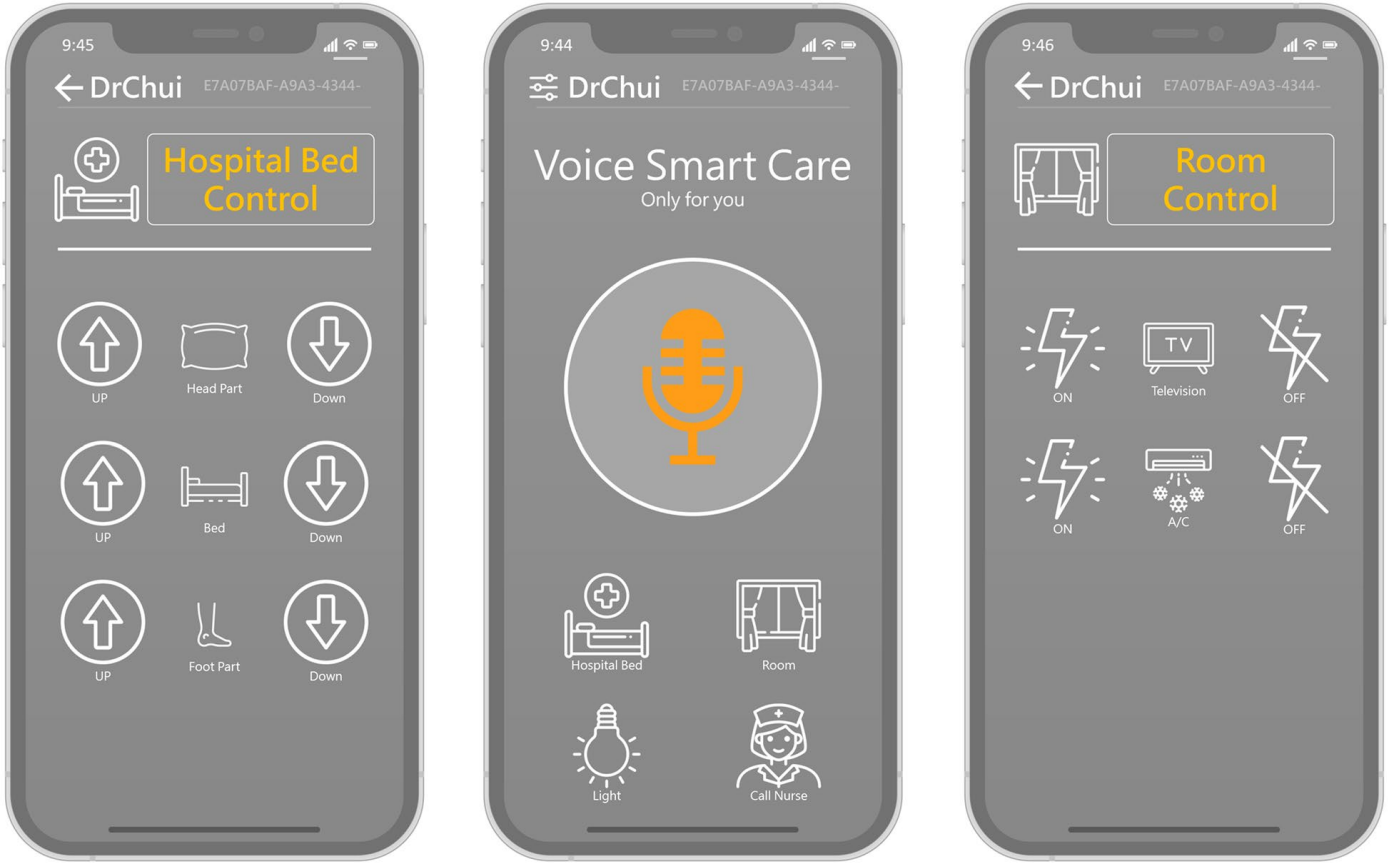
The graphical user interface (GUI) of the VSC system

The Swift programming language was used to build the graphical user interface (GUI) of the VSC system for the IOS system. By contrast, the Java programming language was used to create the Android system. The GUI is shown in Fig. 2. Users could tap the orange button in the middle and speak commands to control facilities in the ward directly. If they are not willing to speak, he/she can also use four buttons below to control the corresponding facilities. In this way, the dominance of facilities in wards can be concentrated on the mobile device, allowing patients to complete the instruction through their own devices. The VSC system was available in both Chinese and English.

### Technology Acceptance Model

**Fig. 3:**
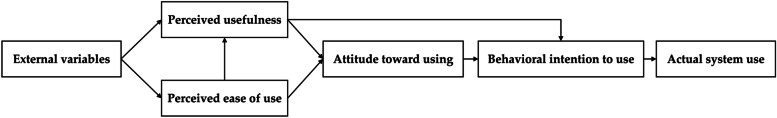
The architecture of the Technology Acceptance Model (TAM)

The technology acceptance model (TAM), a set of theories developed by Fred Davis in 1989, is a better way to explain why people accept or reject computers, especially for technology use behavior [8]. TAM was based on the theory of rational action, which was widely used in the prediction and interpretation of the acceptance behavior of personal information systems. User attitude, mainly influenced by the perceived usefulness (benefit from using the technology) and perceived ease of use (feel free of effort when using the technology), was an essential factor that influenced user behavior (actual usage), and finally decided the acceptance of the information system by the users in the end. Perceived ease of use has a positive effect on perceived usefulness; both perceived ease of use and perceived usefulness affected the attitude toward using, ultimately affected behavioral intent to use and the use of information systems (actual systems) (Fig. 3).

**Fig. 4:**
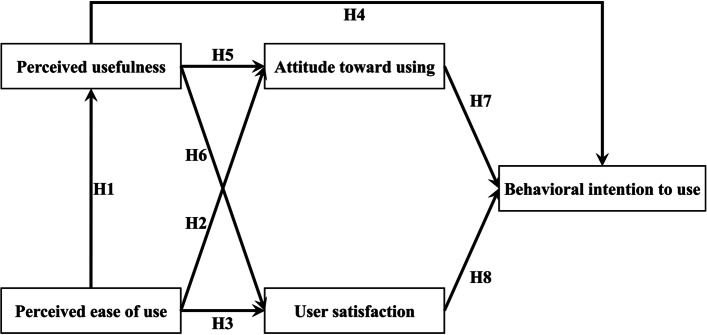
The hypotheses for this study

According to the TAM in literature verification, we assessed the feasibility factors (construct) for the impact of a VSC system with perceived ease of use, perceived usefulness, attitude toward using, user satisfaction, and behavioral intention to use (Fig. 4). As a basis to verify the research structure, the following eight hypotheses were proposed in this study:

H1: "*Perceived ease of use*" has a positive effect on "*Perceived usefulness*".

H2: "*Perceived ease of use*" has a positive effect on "*Attitude toward using*".

H3: "*Perceived ease of use*" has a positive effect on "*User satisfaction*".

H4: "*Perceived usefulness*" has a positive effect on "*Behavioral intention to use*".

H5: "*Perceived usefulness*" has a positive effect on "*Attitude toward using*".

H6: "*Perceived usefulness*" has a positive effect on "*User satisfaction*".

H7: "*Attitude toward using*" has a positive effect on "*Behavioral intention to use*".

H8: "*User Satisfaction*" has a positive effect on "*Behavioral intention to use*".

### Questionnaire Design and Validation

The structural questionnaire adopting TAM was used as a research tool in this study. Before implementing the formal questionnaire, five experts were invited to review the questionnaires (Appendix file). We used HTMT (heterotrait-monotrait ratio) statistics to evaluate convergent and divergent validity between different constructs, which indicate discriminant validity while the value is lower than 0.9 [9]. The Cronbach α value is 0.94, which is above 0.70, suggesting internal consistency reliability [10]. There were two parts to the questionnaire. The first part involved the basic information of the study object. The second part involved TAM, which included perceived usefulness, perceived ease of use, attitude toward using, behavioral intention to use, and user satisfaction. The Likert scale (strongly disagree—1; disagree—2; neutral—3; agree—4; and strongly agree—5) was used to assess the degree of agreement or disagreement [11].

### Statistical Analysis

Pearson's correlation (r) was used to analyze the correlation between research variables. A value of + 1 is a positive linear correlation, 0 is no linear correlation, and − 1 is a negative linear correlation. It means strong correlation, moderate correlation, and weak correlation when the absolute value of *r* = 1.00 ~ 0.70, 0.69 ~ 0.40, and below 0.39, respectively [12]. Multiple regression analysis was used to explore the relationship between one dependent variable and two or more independent variables in four different models [13]. The variance inflation factor (VIF) was used as an indicator of multicollinearity, of which less than ten was considered acceptable [14]. We used Statistical Package for the Social Sciences (SPSS version 24.0; IBM Corp, Armonk, New York) to perform all statistical analyses.

## Results

A total of 32 questionnaires were sent out during the study period. In addition, two invalid questionnaires were excluded because of incomplete filling, no answering, or all options were unchanged. Finally, 30 valid questionnaires remained in our study.

### Demographic Characteristics

In order to understand the basic personal information of the object, relative frequency distribution and percentage were used to describe the "personal background information". For all the patients, the respondents’ basic information included gender, age, education, major language, the cell phone operating system, the daily use frequency of the VSC, and the reason why they don't want to use the VSC system (Table 1). Of the 30 respondents, 17 (57.7%) were aged 21–30 years, 12 (40.0%) were aged 31–40 years, and 1 (3.0%) was aged 41 years or older. More than half (60%) had a bachelor’s degree or above regarding the highest education level.

**Table 1:** Demographic characteristics of the respondents (*N* = 30)

Characteristics	N	%
**Gender**		
Male	15	50.0
Female	15	50.0
**Age (years)**		
21 ~ 30	17	56.7
31 ~ 40	12	40.0
41 or older	1	3.3
**Highest education level**		
High school education or lower	2	6.7
High school graduate	10	33.3
Bachelor’s degree	15	50.0
Master’s degree or above	3	10.0
**Major language**		
Chinese	30	100.0
**The cell phone operating system**		
IOS (iPhone)	10	33.3
Android	20	66.7
**The daily use frequency of the voice smart care system**		
Never used	0	0
1 ~ 5 times	19	63.3
6 ~ 10 times	8	26.7
11 ~ 15 times	0	0
16 ~ 20 times	3	10.0
**The reason why people don’t want to use the voice smart care system** *(Multiple answers, up to three, respondents only need to answer if they select "Never used" or "1* ~ *5 times" in the previous question)*		
Physical discomfort	3	10.7
Too troublesome to use	2	7.1
Poor speech recognition	9	32.0
Mood influence	1	3.6
Not needed	5	17.9
System installation was not easy	1	3.6
Other alternative equipment (e.g., light switch, remote control)	4	14.3
Other reason	3	10.7

Regarding the cell phone operating system, among the 30 respondents, one-third (33.3%) respondents were using IOS. Regarding the daily use frequency of the voice smart care system, of the 30 respondents, 19 (63.0%) respondents used the system 1 ~ 5 times, 8 (26.7%) respondents used the system 6 ~ 10 times, 3 (10.0%) respondents used the system 16 ~ 20 times. Regarding why people don’t want to use the voice smart care system, most respondents reported that the speech recognition quality was not good, followed by they are not needed.

We also provide an open-ended section in the questionnaire to receive how the voice smart care system could be improved. Some users suggested that the system latency should be shorter and the accuracy of voice recognition should be increased. The user also mentioned the system needs to add the usage demonstration, dialect voice control version, and more controllable facilities.

### Measurement Model

We used a 5-point Likert scale to evaluate the degree of agreement or disagreement for each question in the different constructs, which the result was shown in Table 2. There was a tendency for respondents to select agree and strongly agree while filling the questionnaires. However, the reverse coded questions B3 and C4 received the most number of disagree (*N* = 20). We reversed them by following rules before the next step: strongly disagree, disagree, neutral, agree, strongly agree attracted a score of 5, 4, 3, 2, 1, respectively.

**Table 2:** The properties of constructs and questions

Constructs and belong questions	Received numbers of each option				
**Construct I. Perceived usefulness**	**V**	**IV**	**III**	**II**	**I**
A1. I think it is quite helpful for me when using the voice smart care system	10	14	5	1	0
A2. I think it is available to improve my hospitalized quality when using the voice smart care system	8	13	8	1	0
A3. I think using the voice smart care system can quickly operate the equipment in the wards	9	16	5	0	0
A4. I think using the voice smart care system can shorten the time when waiting for medical staff	8	11	9	2	0
A5. I think using the voice smart care system can simplify the operation of equipment in the ward	11	12	6	1	0
A6. Overall, I think the practicability of the voice smart care system is quite high	8	11	9	2	0
**Construct II. Perceived ease of use**	**V**	**IV**	**III**	**II**	**I**
B1. I think it is easy to use the voice smart care system to operate the equipment in the wards	10	18	2	0	0
B2. I don’t think it takes too much effort to learn how to use the voice smart care system	11	18	1	0	0
B3. I need to spend more time than expected to understand how to properly operate the voice smart care system	1	3	4	20	2
B4. I think learning how to operate the voice smart care system is a piece of cake for me	10	20	0	0	0
B5. Overall, I think it’s easy to use the voice smart care system	9	21	0	0	0
**Construct III. User behavior**	**V**	**IV**	**III**	**II**	**I**
C1. I think the voice smart care system is helpful to me	7	16	6	1	0
C2. I am willing to use the voice smart care system	8	16	5	1	0
C3. I think it is positive for the hospital to import the voice smart care system	10	16	4	0	0
C4. I think it is not appropriate to use the voice smart care system	0	1	4	20	5
C5. Overall, I think the advantage of the voice smart care system is more than the disadvantages	9	17	4	0	0
**Construct IV. Attitude toward using**	**V**	**IV**	**III**	**II**	**I**
D1. I think it is worthy of using the voice smart care system	10	19	1	0	0
D2. Because the voice smart care system is helpful to me, I am willing to spend more time understanding how to use it	10	16	4	0	0
D3. I would recommend other people to use the voice smart care system	9	20	1	0	0
D4. In the future, I am willing to use the voice smart care system continuously	9	18	3	0	0
**Construct V. User satisfaction**	**V**	**IV**	**III**	**II**	**I**
E1. I am satisfied with the way the voice smart care system is used	6	18	5	1	0
E2. I am satisfied with the function provided by the voice smart care system	6	16	5	3	0
E3. I think using the voice smart care system can improve my satisfaction with the hospital	10	15	5	0	0
E4. Overall, I am satisfied with the voice smart care system	9	15	6	0	0

Noted: V = strongly agree(5); IV = agree(4); III = neutral(3); II = disagree(2); I = strongly disagree(1)

Table 3 provides the descriptive statistics, validity measurement result, and the values of Cronbach’s α coefficient for each constructed variable. Compared to the mean values among these five constructs, perceived usefulness (PU) ranked the lowest with a score of 3.99 out of 5.00. Meanwhile, respondents’ attitude toward using (AT) of VSC was the strongest, with a score of 4.24 overall. Concerning perceived ease of use (PEOU) to the system, this construct had the second highest-ranked score of 4.18.

**Table 3:** Descriptive statistics and correlation between each pair of construct variables

Constructs	PU	PEOU	AT	BI	US
Correlation coefficient					
** PU**	1				
** PEOU**	0.49^**^	1			
** AT**	0.73^**^	0.53^**^	1		
** BI**	0.71^**^	0.54^**^	0.83^**^	1	
** US**	0.73^**^	0.51^*^	0.64^**^	0.53^**^	1
HTMT statistics					
** PU**	-				
** PEOU**	0.59	-			
** AT**	0.82	0.61	-		
** BI**	0.76	0.61	0.92	-	
** US**	0.81	0.62	0.73	0.58	-
**Number of questions (N)**	6	5	5	4	4
** Range**	3.83—4.10	3.67 – 4.33	4.20—4.30	3.97 – 4.20	3.83 – 4.17
** Mean**	3.99	4.18	4.24	4.07	4.02
** Cronbach's α**	0.91	0.84	0.96	0.86	0.88

Note: *PU* Perceived usefulness, *PEOU* Perceived ease of use, *BI* Behavioral intention to use, *AT* Attitude toward using, *US* User satisfaction

^**^The correlation is significant at a significance level *p* < 0.01

Cronbach’s α analysis was used to measure the reliability among the questionnaire items. Based on the analysis, the internal consistency for each construct was greater than the minimum acceptable level of 0.7, indicating that the survey instrument was reliable and well-constructed. Some constructs like perceived usefulness (PU) and attitude toward using (AT) had an excellent internal consistency as their α coefficients were greater than 0.9.

The HTMT statistics analysis result showed that most constructs had a good discriminant validity (< 0.82) between each other. However, the discriminant validity between attitude toward using(AT) and behavioral intention to use(BI) reached a value of 0.92, which indicated that their concept is similar.

Prior to the multiple linear regression analysis, we evaluated the relationship among five research constructs. When the correlation was at a significance level of 0.01 (two-tailed), there were strong positive correlations between attitude toward using (AT) and behavioral intention to use (BI) (*r* = 0.83, *p* < 0.01), perceived usefulness (PU) and attitude toward using (AT) (*r* = 0.73, *p* < 0.01), perceived usefulness (PU) and user satisfaction (US) (*r* = 0.73, *p* < 0.01), perceived usefulness (PU) and behavioral intention to use (BI) (*r* = 0.71, *p* < 0.01).

### Hypothesis Testing

Table 4 showed the coefficients of multiple regression analysis in four different models, and auxiliary regression reported that there were no collinearity problems (Model 1: VIF = 1.00 < 10; Model 2: VIF = 1.32, 1.33 < 10; Model 3: VIF = 1.33, 1.32 < 10; Model 4: VIF = 2.85, 2.29, 2.22 < 10) among them. Each model is described as follows:

**Table 4:** The results of multiple regression analysis

Model		Unstandardized Coefficients		Standardized Coefficients	t	Sig *p* values	VIF
		**B**	**Std. Error**	**Beta**			
**1**	(Constant)	1.01	1.01		0.99	.33	
	Perceived ease of use (H1)	0.71**	0.24	0.49	2.97	< .01	1.00
	Dependent variable: *Perceived usefulness* (*R* = 0.49; *R*^*2*^ = 0.24; adjusted *R*^*2*^ = 0.21; *F* = 8.82, *p* < .001)						
**2**	(Constant)	0.96	0.64		1.45	.16	
	Perceived ease of use (H2)	0.26	0.17	0.22	1.53	.14	1.32
	Perceived usefulness (H5)	0.51***	0.12	0.63	4.36	< .001	1.33
	Dependent variable: *Attitude toward using* (*R* = 0.76; *R*^*2*^ = 0.58; adjusted *R*^*2*^ = 0.54; *F* = 18.32, *p* < .001)						
**3**	(Constant)	0.49	0.76		0.65	.52	
	Perceived ease of use (H3)	0.28	0.20	0.20	1.38	.18	1.33
	Perceived usefulness (H6)	0.59***	0.14	0.63	4.26	< .001	1.32
	Dependent variable: *User satisfaction* (*R* = 0.75; *R*^*2*^ = 0.56; adjusted *R*^*2*^ = 0.52; *F* = 16.93, *p* < .001)						
**4**	(Constant)	0.94	0.44		2.15	.04	
	Perceived usefulness (H4)	0.24	0.14	0.29	1.68	.11	2.85
	Attitude toward using (H7)	0.69***	0.16	0.70	4.49	< .001	2.29
	User satisfaction (H8)	-0.12	0.13	-0.14	-0.88	.36	2.22
	Dependent variable: *Behavioral intention to use* (*R* = 0.85; *R*^*2*^ = 0.72; adjusted *R*^*2*^ = 0.69; *F* = 22.48, *p* < .001)						

Note: *VIF* Variance inflation factor; *significant at *p* < .05; **significant at *p* < .01; ***significant at *p* < .001

In the first model, we explored the factor (perceived ease of use) that influences users to think it is beneficial while adopting the system. The perceived ease of use with statistical significance (*F* = 8.82, *p* < 0.001) could effectively explain 21% (R2 = 0.21) of the overall variance, and had a positive and statistically significant effect by perceived ease of use (β = 0.49, t = 2.97, *p* < 0.01) which supported H1.

In the second model, we explored the factor (perceived ease of use & perceived usefulness) that influences users' assessment of using the specific system. The two independent variables could effectively explain the 54% (R2 = 0.54) of the overall variance with statistical significance (*F* = 18.32, *p* < 0.001). The perceived usefulness had a positive and statistically significant effect on attitude toward using (β = 0.63, t = 4.36, *p* < 0.001), which supported H5. However, there was no significant correlation between perceived ease of use and attitude toward using (*p* = 0.14 > 0.01). Thus, H2 was not supported. It means that the user’s evaluation of the system depends more on whether the system can provide substantial help instead of it is easy to use or not.

In the third model, the influencing factors (independent variables) were the same as Model 2 but with different dependent variables (user satisfaction). Two independent variables could effectively explain the 52% (R2 = 0.52) of the overall variance with statistical significance (*F* = 16.93, *p* < 0.001). The perceived usefulness had a positive and statistically significant effect on user satisfaction (β = 0.63, t = 4.26, *p* < 0.001), which supported H6. However, there was no significant correlation between perceived ease of use and user satisfaction (*p* = 0.18 > 0.01). Thus, H3 was not supported.

In the fourth model, we evaluated factors (perceived usefulness, attitude toward using, and user satisfaction) that affected behavioral intention to use. The three independent variables could effectively explain the 69% (R2 = 0.69) of the overall variance with statistical significance (*F* = 22.48, *p* < 0.001). The attitude toward using had a positive and statistically significant effect on behavioral intention to use (β = 0.70, t = 4.49, *p* < 0.001), which supported H7. However, there were no significant correlations between perceived usefulness and behavioral intention to use (*p* = 0.11 > 0.01), and between user satisfaction and behavioral intention to use (*p* = 0.36 > 0.01). Thus, H4 and H8 were not supported. Since their concepts are highly similar, we were not surprised by this result.

**Fig. 5:**
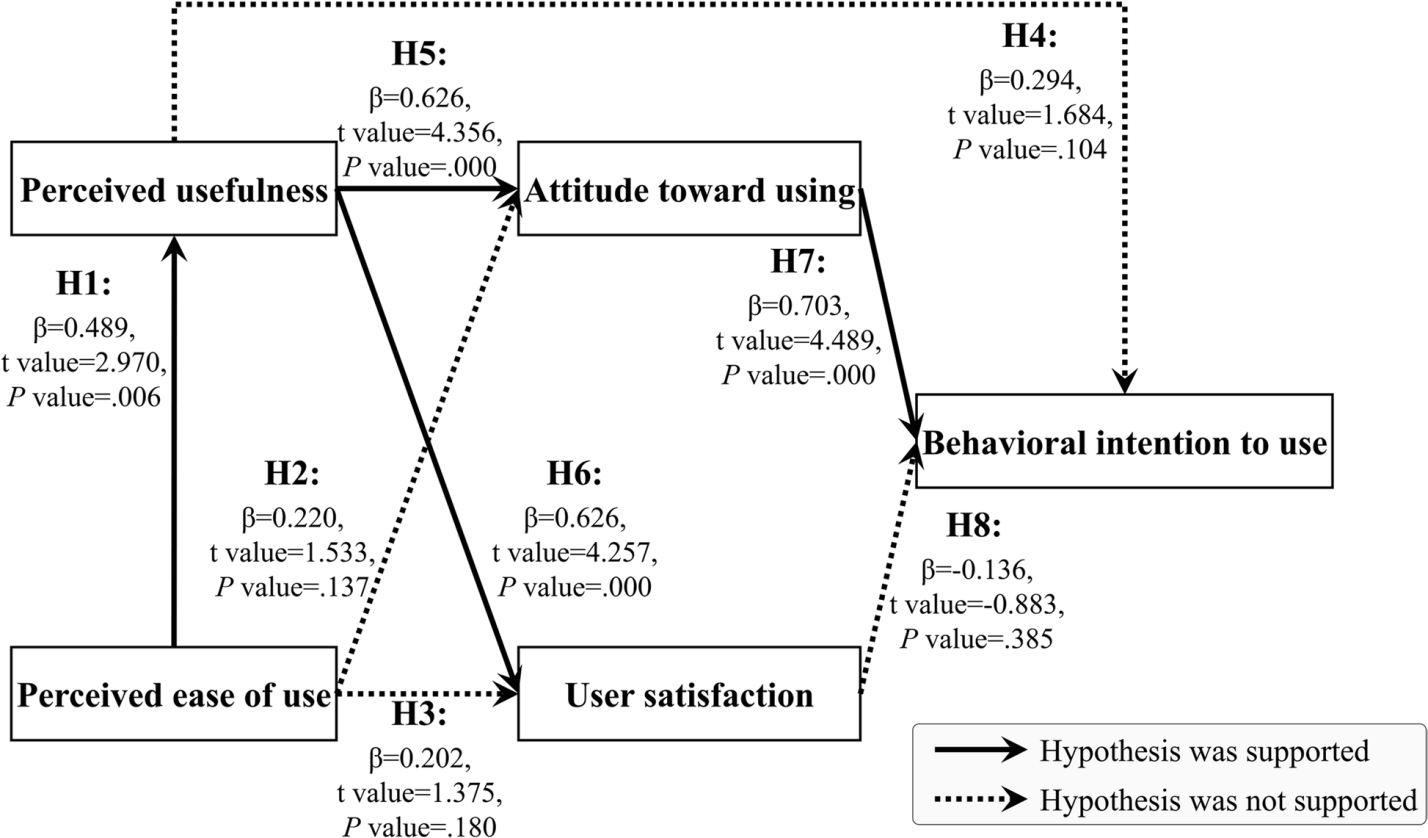
Results for hypothesis analysis

Based on the above research results, the eight hypotheses of this research could be verified, and the results were summarized in Fig. 5. We could find out that the system could provide practical help (perceived usefulness) is the crucial factor determining users' willingness and satisfaction (attitude toward using and user satisfaction) which is driven by whether easy to operate (perceived ease of use).

## Discussion

This study implemented the VSC, a voice-based control system in the hospital wards, and evaluated patients’ acceptance of the system through structural questionnaires after practical use of VSC for more than two days. Many researchers have been studying the usability of IoT in smart hospitals [15–17]. In this study, we used the TAM to qualitatively explore user acceptance for a voice-based control system among hospitalized patients. The constants included perceived ease of use, perceived usefulness, attitude toward using, user satisfaction, and behavioral intention to use. The result generated from our study could provide valuable insights when hospitals plan to implement a similar system in their wards, ultimately to improve patient satisfaction.

Furthermore, we used the TAM to determine the constants which affect user acceptance. Our result also indicated that three TAM constants (perceived ease of use, perceived usefulness, and use willingness) were crucial to the tendency of people when using the VSC or any other intelligent control system, which complied with the findings from other studies [18, 19].

Perceived ease of use, a person who believes that using a particular system would be free from effort [8], was an important factor that affects the perceived usefulness. According to the question “The reason why I don’t want to use the voice smart care system”, poor speech recognition was the most frequently reported answer among the respondents. Intuitively, the poor speech recognition quality increased the difficulty while using the system, making users spend more time completing their tasks than initially expected [20]. However, our results showed that perceived ease of use was not a determinant of attitude toward using, consistent with past studies [21, 22].

Perceived usefulness, a person who believes that using a particular system would enhance their job performance [8], was a crucial predictor by the past study [23]. Having the same result from our study, perceived usefulness positively affected both attitudes toward using and user satisfaction, which is promoted by the perceived ease of use. Thus, this auxiliary system should be useful to those in need, such as postoperative patients or disabled individuals. The VSC system allows patients to control facilities without assistance. Providing substantial help will make users have a positive attitude and satisfaction while using the new technology.

The VSC system allows patients to control facilities in the wards by speaking commands to their mobile phones or tablets and has a high acceptance rate in our study. We believe that there are some potential opportunities to implement the analogous smart healthcare system in wards. Therefore, based on our research results, the suggestions for hospitals when designing a voice-based control system were as given below. First, since perceived usefulness was a major factor that decided the patient's satisfaction (H5) and attitudes (H6), ultimately whether adopted the system or not. The routine control tasks should be completed more efficiently and make more facilities controllable (e.g., air conditioner). Second, users should start intuitively without relying on a manual book since users only consider that system is useful under the premise of being easy to operate. Third, the comments and feedback given by the actual users of the system are crucial for other potential adopters to start using the system [24]. Having a satisfactory user experience at the beginning will be of advantage to promote the system in the future. By following these design guidelines for the voice-based control system, patient autonomy may be improved [25, 26], which could decrease medical staff burnout [27] and provide possible benefits to patient safety at the same time.

### Limitations

This study has several limitations. First, the generalizability of this study could be limited by its low number of participants. Since only two wards were reconstructed as our experimental field, our ability to collect data is restricted. Second, some of the data patients provided were rely on their remembrance. A more reliable data collection way such as system log files should be used. Third, participants are relatively young (age < 45) because the study was conducted in general pediatric wards. The elderly people’s acceptance of the VSC system should also be studied. Four, the patient's condition was not included in the basic demography of the respondent. For patients with any mobility problems, this information should be stated in future studies. Lastly, the viewpoints of medical staff should also be evaluated. Our research only focused on the patients’ point of view, but the medical staff's opinion was also important. Multifaceted evaluation can make the system more comprehensive, which could genuinely reduce the medical staff's burden.

## Conclusion

We have demonstrated a solution to develop the VSC, a voice-based control system to interact with equipment in the ward. Our experience could potentially provide other hospitals while implementing a similar system. We also explored the key factors on patient acceptance of the system through TAM. The results showed that perceived usefulness was determined as a significant factor to impact the attitude toward using and user satisfaction, which means the system function should precisely meet the patients' demand. Additionally, a clever system design is important since perceived ease of use positively affects perceived usefulness. These results could expand the functionality of the hospital’s traditional ward control system and shed light on the implementation of the voice-based control system.

## Supplementary Information

**Additional file 1.**

## Data Availability

The datasets used and/or analysed during the current study are available from the corresponding author on reasonable request.

## Abbreviations

VSC: Voice smart care
TAM: Technology acceptance model
I-CVI: Content validity index
S-CVI: Scale-level content validity index
SPSS: Statistical package for the social sciences
VIF: Variance inflation factor
