# Supplementary material for: Voice-based control system for smart hospital wards: a pilot study of patient acceptance
Source: BMC Health Serv Res. 2022 Mar 3;22:287. doi: 10.1186/s12913-022-07668-1 (PMC8892698; doi:10.1186/s12913-022-07668-1)
Supplement: Supplementary file 1 — Additional file 1. [file 12913_2022_7668_MOESM1_ESM.docx]

Appendix/Supplementary

**Research questionnaire**

Dear Sir/Madam:

This is an academic questionnaire that evaluates

your acceptance of the voice smart care system in the ward after actual use. Please answer the following questions according to your own feelings.

**The "voice smart care system" allows the patient to use smartphones or tablet computers to operate the facilities in the ward, including beds, lights, TVs, curtains, etc., through the voice or touch function of the device. This system allows patients to control the ward equipment without other people's help.**

The results of this study are for academic purposes only. The information which you provide will be absolutely confidential. Please rest assured to answer the questions. Your kindly support and cooperation will be the key factor for completing this research. Thank you very much for spending the time to finish the questionnaire.

Best regards.

**【Term definition Voice smart care system】**

Patients use smartphones or tablet computers to operate the facilities in the ward, including beds, lights, TVs, curtains, etc., through the voice or touch function of the device. The voice smart care system allows patients to control the ward equipment without other people's help.

**【Construct I. Perceived usefulness】**

Patients believe that using the voice smart care system can provide essential assistance for themselves, and they have an affirmative attitude towards this system.

**【Construct II. Perceived ease of use】**

Patients believe that the voice smart care system is easy to learn and use. Generally, perceived ease of use will directly affect user behavior and attitude toward using.

**【Construct III. User behavior】**

The personal preference of voice smart care system for the patients, who have positive or negative comments on the system.

**【Construct IV. Attitude toward using】**

Patients would like to use the voice smart care system under the situation of having self-awareness in the future.

**【Construct V. User satisfaction】**

The patients' satisfaction with the system after using a voice smart care system. It will directly affect user behavior and attitude toward using.

**【Part One】Questionnaire Content**

The following multiple-choice questions with a single answer have "Strongly Agree", "Agree", " Neutral ", "Disagree", and "Strongly Disagree" five options. Please choose the closest answer identical to your own feelings according to the description of the question.

| **Construct I. Perceived usefulness** | | | | | |
| --- | --- | --- | --- | --- | --- |
|  | Strongly Agree | Agree | Neutral | Disagree | Strongly Disagree |
| A1. I think it is quite helpful for me when using the voice smart care system. |  |  |  |  |  |
| A2. I think it is available to improve my hospitalized quality when using the voice smart care system. |  |  |  |  |  |
| A3. I think using the voice smart care system can quickly operate the equipment in the wards. |  |  |  |  |  |
| A4. I think using the voice smart care system can shorten the time when waiting for medical staff. |  |  |  |  |  |
| A5. I think using the voice smart care system can simplify the operation of equipment in the ward. |  |  |  |  |  |
| A6. Overall, I think the practicability of the voice smart care system is quite high. |  |  |  |  |  |

| **Construct II. Perceived ease of use** | | | | | |
| --- | --- | --- | --- | --- | --- |
|  | Strongly Agree | Agree | Neutral | Disagree | Strongly Disagree |
| B1. I think it is easy to use the voice smart care system to operate the equipment in the wards. |  |  |  |  |  |
| B2. I don’t think it takes too much effort to learn how to use the voice smart care system. |  |  |  |  |  |
| B3. I need to spend more time than expected to understand how to properly operate the voice smart care system. |  |  |  |  |  |
| B4. I think learning how to operate the voice smart care system is a piece of cake for me. |  |  |  |  |  |
| B5. Overall, I think it’s easy to use the voice smart care system. |  |  |  |  |  |

| **Construct III. User behavior** | | | | | |
| --- | --- | --- | --- | --- | --- |
|  | Strongly Agree | Agree | Neutral | Disagree | Strongly Disagree |
| C1. I think the voice smart care system is helpful to me. |  |  |  |  |  |
| C2. I am willing to use the voice smart care system. |  |  |  |  |  |
| C3. I think it is positive for the hospital to import the voice smart care system. |  |  |  |  |  |
| C4. I think it is not appropriate to use the voice smart care system. |  |  |  |  |  |
| C5. Overall, I think the advantage of the voice smart care system is more than the disadvantages. |  |  |  |  |  |

| **Construct IV. Attitude toward using** | | | | | |
| --- | --- | --- | --- | --- | --- |
|  | Strongly Agree | Agree | Neutral | Disagree | Strongly Disagree |
| D1. I think it is worthy of using the voice smart care system. |  |  |  |  |  |
| D2. Because the voice smart care system is helpful to me, I am willing to spend more time understanding how to use it. |  |  |  |  |  |
| D3. I would recommend other people to use the voice smart care system. |  |  |  |  |  |
| D4. In the future, I am willing to use the voice smart care system continuously. |  |  |  |  |  |

| **Construct V. User satisfaction** | | | | | |
| --- | --- | --- | --- | --- | --- |
|  | Strongly Agree | Agree | Neutral | Disagree | Strongly Disagree |
| E1. I am satisfied with the way the voice smart care system is used. |  |  |  |  |  |
| E2. I am satisfied with the function provided by the voice smart care system. |  |  |  |  |  |
| E3. I think using the voice smart care system can improve my satisfaction with the hospital. |  |  |  |  |  |
| E4. Overall, I am satisfied with the voice smart care system. |  |  |  |  |  |

**【Part Two】Basic information**

- 1. Gender:

□Male; □Female

- 1. Your age:

□20-40 years old; □40-60 years old; □60 years old or older

- 1. The highest degree you have completed:

□Illiterate; □Elementary school; □Junior high school; □Senior high school;
□Bachelor degree; □Master degree or above

- 1. Major language:

□Chinese; □English; □Other

- 1. The brand of cellphones:

□iPhone; □HTC; □Sony; □OPPO; □Samsung; □Xiaomi; □Redmi; □Asus;
□Other

- 1. The daily use frequency of the voice smart care system:

□Never used; □1~5 times; □6~10 times; □11~15 times; □16~20 times

- 1. The reason does NOT want to use the voice smart care system (Multiple answers, up to three, only need to answer if select "Never used" or "1~5 times" in the previous question.):

□Physical discomfort; □Too troublesome to use; □Poor speech recognition;
□Mood influence; □No needed; □System installation was not easy;
□System function did NOT meet requirements;
□Other alternative equipment (e.g., light switch, remote control); □Other reason
